# Reliability of magnetoencephalography beta desynchronization for language lateralization in a subsequent memory effect paradigm

**DOI:** 10.1016/j.cnp.2026.06.005

**Published:** 2026-06-12

**Authors:** Emilie Müller, Paulina Riegler Vergara, Nadia Müller-Voggel, Martin Kaltenhäuser, Arnd Doerfler, Hajo Hamer, Daniel Delev, Oliver Schnell, Stefan Rampp

**Affiliations:** a Department of Neurosurgery, Universitätsklinikum Erlangen, Friedrich-Alexander-Universität Erlangen-Nürnberg (FAU), Erlangen, Germany; b Department of Neuroradiology, Universitätsklinikum Erlangen, Friedrich-Alexander-Universität Erlangen-Nürnberg (FAU), Erlangen, Germany; c Department of Neurology, Epilepsy Center, Universitätsklinikum Erlangen, Friedrich-Alexander-Universität Erlangen-Nürnberg (FAU), Erlangen, Germany; d Department of Neurosurgery University Hospital Halle (Saale), Germany; e Full Member of ERN EpiCARE, Germany

**Keywords:** Language lateralization, MEG, SME, Surgery

## Abstract

**Objective:** Verb generation is associated with significant lateralized beta desynchronization in language but also motor related areas. Subsequent Memory Effect (SME) paradigms comprise receptive language and memory tasks. The current study examined whether lateralized beta patterns in language and/or motor related areas can be obtained with the receptive SME paradigm.

**Methods:** MEG was recorded in 20 healthy right-handed adults during encoding and recognition phases of a word SME task. Measurements were repeated after 7–10 days to assess reliability. Post-stimulus beta (13–35 Hz) decrease was analyzed, and lateralization indices (LI > 0.1 indicating left dominance) were evaluated. Regions of interest (ROI) included IFG pars opercularis and triangularis (IFGO, IFGT), supramarginal gyrus, angular gyrus, superior temporal gyrus, and precentral gyrus (PreCG). An exploratory analysis included additional AAL regions.

**Results:** Significant beta desynchronization within the ROIs was observed in 65–90% of participants. Lateralization was successful in up to 79% in the IFGT during encoding, 76% during recognition and 100% in the IFGO in encoding-recognition concordant cases. Exploratory analyses showed strong left lateralization in frontal, temporal and especially parietal areas. Initial reliability was limited, yet within-session concordance showed robust dominance with up to 100% concordance. Significant lateralized beta desynchronization was observed in the PreCG in 70–90% of participants.

**Conclusion:** The SME paradigm shows promise in healthy participants, warranting investigation in patients with atypical language organization.

**Significance:** Language related beta desynchronization is also observed in a receptive SME-task. Lateralized beta desynchronization in motor areas however suggest their contribution in verbal working memory and semantic control.

## Highlights

• Language memory task enables MEG language lateralization using beta-band decreases
• Motor-area beta decreases in receptive task suggest role in cognitive control process
• Recording of encoding and recognition enhances reliability of language lateralization

## 1 Introduction

In the treatment of numerous conditions affecting the brain, such as tumors or pharmacoresistant epilepsy, surgical intervention is a viable or even the preferred therapy option. However, this approach is not without risks, including potential adverse effects such as functional deficits, e.g. of speech (Tebo et al., 2014).In particular, patients undergoing surgical procedures in the vicinity of areas related to language, such as the anterior temporal lobe or the inferior frontal gyrus in the language-dominant hemisphere, may experience a decline in their language skills following surgical intervention (Rugg-Gunn et al., 2020). To avoid these outcomes, presurgical lateralization and localization of language is of utmost importance and the search of new methods to supplement or preferably replace invasive procedures, such as the Wada test, has been ongoing for decades (Papanicolaou et al., 2018).

Magnetencephalography (MEG) has played an important role in this search since its inception (Hämäläinen et al., 1993). It is a non-invasive procedure that allows for the recording of neuronal activation by measuring changes in the associated magnetic fields (Papanicolaou et al., 2004). It exhibits exceptional temporal and high spatial resolution, with the latter dependent upon signal-to-noise ratio (Hansen et al., 2010). Computer algorithms are employed to calculate the origin of the activity, a process known as source imaging (Hämäläinen et al., 1993; Hansen et al., 2010). In contrast to fMRI, which is contingent upon blood (de)oxygenation and cerebral blood flow, MEG is not heavily influenced by tumor-induced alterations of vascularization. MEG is therefore an appropriate modality for individuals with tumors or other malformations (Kamada et al., 2007). MEG records neural activity in a passive manner (Hansen et al., 2010), is non-invasive, virtually risk-free and induces only minimal discomfort (Papanicolaou et al., 2004; Pirmoradi et al., 2010). Therefore, this approach can be employed at early stages of the pre-operative workup process (Kreidenhuber et al., 2019).

Between the results of MEG and the Wada procedure for lateralization of the language dominant hemisphere, a high level of concordance has been shown; moreover MEG provides accurate data regarding the location of receptive and expressive language cortex (Papanicolaou et al., 2004; Hirata et al., 2010; Tanaka et al., 2013; Papanicolaou et al., 2019; Herfurth et al., 2022; Noorizadeh et al., 2024). These studies, however, also show the main drawback of MEG language mapping, namely its limited specificity, which leads to overestimation of areas involved in language processing (Janecek et al., 2013; Hirata et al., 2010; Papanicolaou et al., 2014). However, alternative methods, including TMS, fMRI and direct cortical stimulation, have also been shown to demonstrate similar limitations (Papanicolaou et al., 2014; Ille et al., 2015).

These issues are particularly relevant in patients with atypical language lateralization, which refers to those with either complete right-sided dominance or bilateral involvement. In these cases, conflicting results have been observed in a significant proportion of cases (Janecek et al., 2013). Consequently, there is still a need for the development of methods that enable reliable and precise language lateralization at the level of the individual patient.

In a previous study, we explored beta desynchronization as a marker of language lateralization during a verb generation (VG) paradigm and compared the results to fMRI recordings and the Wada test (Herfurth et al., 2022). The term “Beta desynchronization” encompasses a range of mechanisms which result in a reduction of beta power in comparison to a baseline condition. Next to desynchronization of neuronal populations, reduction of neuronal activity itself, change of phase shifts or loss of coherence of potentially distinct populations leads to a reduction of beta power on the surface, as detected by MEG. Here, we do not distinguish between these mechanisms and generally address the observation of reduced beta power vs. baseline.

In conventional MEG analysis, broadband frequencies ranging from 1 to over 50 Hz are taken into account, as the overall activation is considered to determine the language dominant hemisphere (Tanaka et al., 2013). However, broadband ranges contain delta, theta and gamma frequencies which are related to excitatory activity and alpha and beta frequencies which are related to idling and inhibitory activity (Giraud and Poeppel, 2012; Weiss and Mueller, 2012). Our study showed that involvement in processing tasks is associated with a decrease in the beta frequency bands, exhibiting a high degree of concordance with the invasive Wada test and demonstrating superior performance relative to fMRI (Herfurth et al., 2022).

In light of these promising results, the current study aims to investigate beta desynchronization during a Subsequent Memory Effect (SME) paradigm using word stimuli and to evaluate its effectiveness for reliable lateralization of language. VG is dominated by (covert) language production. The associated beta desynchronization has been observed not only in language related areas, but also in precentral regions. This finding has been related to activation of early motor programs preparing (but not executing) overt language production (Herfurth et al., 2022). In contrast, SME as implemented here, mainly utilizes language reception and recognition, mostly lacking any elements of language production. Notably, the subsequent memory effect itself refers to more pronounced responses during encoding of stimuli that are later better recognized. The focus of the current study is however to utilize the same paradigm design to evaluate beta desynchronization during the non-expressive, non-motor language task, i.e., not necessarily dependent on successful recognition and thus memory function.

If beta desynchronization is contingent on activation of motor programs, the SME paradigm should not show a relevant reduction of beta in motor-related areas. In contrast, such beta desynchronization should be observable if it plays a role in disinhibition and unmasking of task-relevant systems and if motor systems are also involved in language reception/recognition in the given context. While studies investigating SME proper have shown beta desynchronization in e.g. the inferior frontal gyrus (Meeuwissen et al., 2011; Hanslmayr et al., 2012), it remains unclear whether beta desynchronization can also be observed in motor and/or premotor areas.

Correspondingly, the presented study investigates beta desynchronization during a receptive task as a marker for the language dominant hemisphere both on a group and individual level, as well as its reliability in healthy right-handed persons.

## 2 Material and methods

### 2.1 Participants

Twenty healthy right-handed participants (8 females) were enrolled. Inclusion criteria were: Adult, right-handed, native German speakers with normal or corrected-to-normal vision and MEG compatibility (i.e., no implanted metal or electronic devices). Right-handedness was assessed with the Edinburgh Handedness Inventory (EHI) and ranged from 50 to 100 (mean 84.6)(Oldfield, 1971). The mean age was 23.9 years, ranging from 18 to 35 years. All participants gave their written informed consent prior to the study with a form approved by the ethical board of the Universitätsklinikum Erlangen (registration number 22-70_2-B).

### 2.2 Data acquisition

MEG data were acquired using a 248-channel whole-head system with magnetometers (Magnes 3600 WHS, 4D Neuroimaging, San Diego, CA). The participant coordinate system was established using landmarks (left and right preauricular points, nasion, Cz, inion) marked with a digitizer (Polhemus Fastrak, Colchester, VT, USA). Head coils were positioned at the left and right preauricular points, left and right mastoid, and forehead. Data were sampled at 1017.25 Hz with analog filters set to 1–400 Hz.

### 2.3 Procedure

Participants were recorded in a supine position, with a screen 60 cm above them displaying white words on a black background. Two response devices were placed next to the participants. The SME paradigm was explained to them beforehand.

### 2.4 Subsequent memory effect paradigm

The SME paradigm consists of two phases: encoding and recognition. During encoding, 100 German one- or two-syllable nouns were shown consecutively for 2 s each. After each word, a 1–6 rating scale appeared, asking participants to rate word pleasantness while allowing time for blinking and swallowing. A fixation cross followed for 1–1.5 s. The rating was intended to promote active engagement and was not used for analysis.

**Fig. 1:**
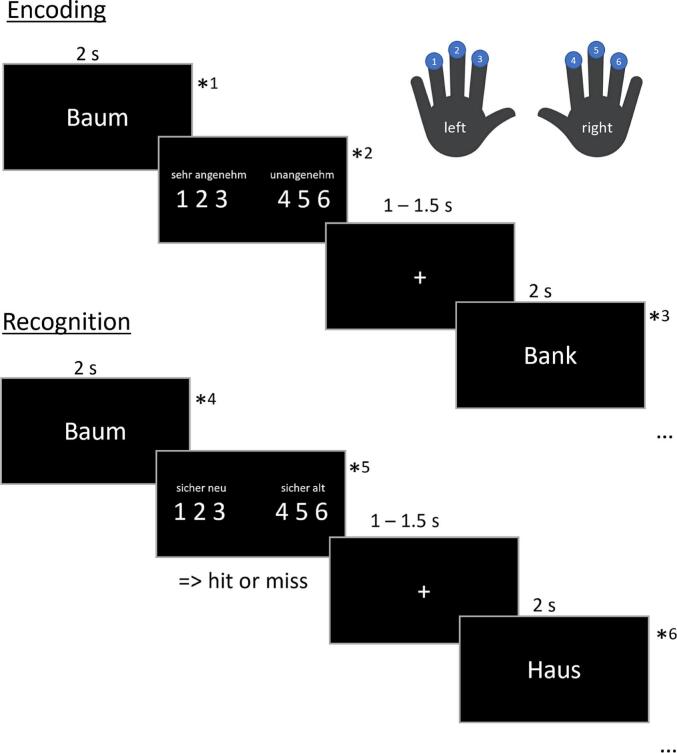
Diagram of our Subsequent Memory Effect paradigm: The SME paradigm comprised an encoding phase (100 German nouns, 2 s each, pleasantness rating on a 1–6 scale, jittered fixation 1–1.5 s) and a recognition phase (150 German nouns, 2 s each, old/new judgment on a 1–6 scale, jittered fixation 1–1.5 s). *^1^ German noun “tree” *^2^ “pleasant: 1 2 3 - unpleasant: 4 5 6” *^3^ German noun “bench” or “bank”. *^4^ German noun “tree” *^5^ “sure that new: 1 2 3 - sure that old: 4 5 6” *^6^ German noun “house”.

After a brief rest to ensure optimal concentration, recognition began. Participants saw 150 German words (100 old, 50 new) in randomized order for 2 s each. Following each word, a 1–6 scale asked participants to indicate whether the word had been seen during encoding, expressing their certainty. A fixation cross appeared for 1–1.5 s before the next word (Fig. 1).

### 2.5 Evaluation of reliability

To assess reliability, each participant repeated the paradigms 7–10 days after the first session. The paradigm was explained before the first session to prevent familiarity from making the second session easier. For each session, one of three different word lists was used. The first and second session used different lists and choice and sequence of the specific list was randomized across participants.

Reliability was further evaluated by calculating the percentage of left lateralization for each ROI across both sessions. Additionally, the localization and lateralization of participants showing identical lateralization in both encoding and recognition phases were compared. We hypothesized that concordant results would indicate robust findings and show significant left-sided lateralization, as all participants were right-handed, healthy individuals.

### 2.6 Analysis

Reaction times and hit rates were examined. For reaction times, the first and third quartiles, median, and maximum were calculated, and mean hit rates were computed. Differences in reaction times and hit rates between sessions were assessed using ANOVA (R Core Team 2024).

Performance was analyzed using a ROC approach, adjusting the rating threshold for a correctly identified word to the individual subjects' behavior. For details see (Hanslmayr et al., 2009).

MEG data were analyzed using MATLAB (R2021a), FieldTrip (20210128), and Brainstorm (22 Nov 2024). Flat or excessively noisy channels and segments with visually identified artifacts were excluded. Cardiac and eye-blink artifacts were removed using Signal-Space Projection (SSP) based on manually selected artifact patterns. Data were then segmented into epochs with a 1000 ms interval before stimulus onset (pre-trigger) and a 1500 ms interval after stimulus onset (post-trigger). Epochs were demeaned using the pre-trigger interval as baseline. A further visual inspection was performed to reject any residual artifacts.

The cleaned data were then submitted to source imaging using a standard single shell volume conductor corresponding to the inner surface of the skull as provided by the fieldtrip toolbox (https://www.fieldtriptoolbox.org/). A MNI-aligned 3d grid with 10 mm resolution constrained to the intracranial space was used as source space. Activity in the beta frequency band (13–35 Hz) was determined in source space using the DICS (dynamic imaging of coherent sources) method (Gross et al., 2001) using 10% regularization, combined for both a baseline (−700 ms to 0 ms relative to stimulus onset) and an activation segment (300 ms to 1000 ms) according to (Herfurth et al., 2022). To this end, data were transformed to the frequency domain by multitaper frequency transformation with discrete prolate spheroidal sequences (DPSS), however using only a single taper with smoothing covering the beta frequency band (24 Hz center frequency, 11 Hz smoothing).

Beta desynchronization, i.e., reduction of beta power in source space in the post-trigger compared to the baseline segment were evaluated statistically by performing non-parametric Monte Carlo permutation tests based on one-tailed dependent-sample *t*-tests with a *p*-value of 0.05 of power values of single trials and 10.000 permutations. The cluster-correction method was applied to account for the type I error rate (Maris and Oostenveld, 2007).

Resulting t-values were parcellated by averaging all t-values with a corresponding p-value <0.05 within each region of the Automatic Anatomical Labeling (AAL) atlas, excluding the cerebellum and the basal ganglia and thalamus (Tzourio-Mazoyer et al., 2002). Note that since one-sided statistical evaluation only considered beta desynchronization, this procedure resulted in all averaged t-values ≤0.

Lateralization indices (LI) were computed as applied in (Herfurth et al., 2022):

$$\left({\mathrm{av}}_{\mathrm{left}}–{\mathrm{av}}_{\mathrm{right}}\right)/\left({\mathrm{av}}_{\mathrm{left}}+{\mathrm{av}}_{\mathrm{right}}\right)\;\left(\mathrm{av}–\mathrm{average}\;t-\mathrm{value of voxels with}\;p<0.05,\mathrm{all}\;\mathrm{av}.\leq 0\right)$$

A LI value of 1.0 indicates complete left lateralization of beta desynchronization, and − 1.0 indicates complete right lateralization. Left-sided dominance was defined as LI > 0.1 (Herfurth et al., 2022).

To assess statistical significance—i.e., the consistency of lateralization within each parcel across participants—bootstrap statistics were applied to generate a random distribution. In each of 1000 randomizations, parcel averages from the left and right were swapped with a probability of 0.5, and LI values were calculated. Actual LI values were then compared to this distribution, z-scored, and converted into *p*-values.

Primary regions of interest (ROIs) were defined based on (Herfurth et al., 2022) and included the IFG pars opercularis (IFGO) and triangularis (IFGT), supramarginal gyrus (SMG), gyrus angularis (ANG), superior temporal gyrus (STG), and precentral gyrus (PreCG). *P*-values within this group p-values were corrected for multiple comparison using FDR-adjustment (Yekutieli and Benjamini, 1999).

A secondary, exploratory analysis included all AAL regions except the cerebellum and subcortical structures. FDR adjustment was not conducted.

## 3 Results

### 3.1 Reaction time and performance

In the first session, all participants reacted within a maximum of 4.94 s (median 0.59; first quartile 0.37 – third quartile 1.09), with an average reaction time of 0.91 (SD 0.83) seconds. Participants performed very well, correctly identifying most words (median 125.5 of 150; first quartile 121.5 – third quartile 132.5). In the second session, reaction times appeared slightly faster (0.52; 0.35–1.04; mean 0.88, SD 0.86), but the difference was not significant (*p* = 0.287). Accuracy, however, decreased significantly, with fewer correctly identified words (113; 103–130.25; hit rate: p 0.0441).

### 3.2 Beta desynchronization

In the encoding condition of the first session, up to 14 (mean 13.7; SD 0.5) of the 20 participants showed significant beta desynchronization compared to pre-trigger baseline in at least one primary ROI, and up to 14 (13.2; 1.0) in at least one investigated AAL parcel. During the recognition phase of the first session, up to 17 (16.7; SD 0.8) of 20 participants showed significant beta desynchronization compared to pre-trigger baseline in at least one primary ROI, and up to 17 (16.2; 1.0) in at least one investigated AAL parcel.

**Fig. 2:**
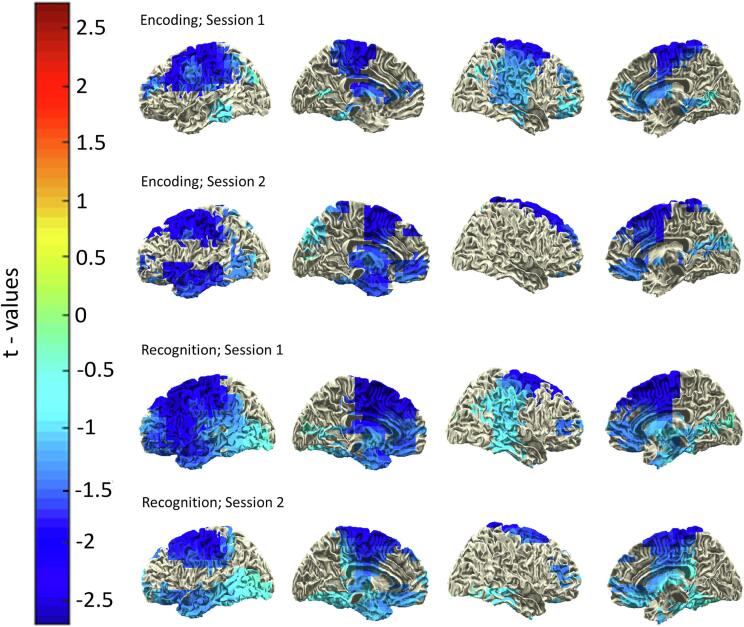
Analysis of beta power in relation to baseline on a group level: Beta power decreased across the cortex, with the strongest decrease in the left hemisphere during both the encoding and recognition phase, primarily in frontal, parietal and temporal areas*.*

During the second session in the encoding condition, up to 18 (17.8; 0.47) participants showed significant beta desynchronization compared to pre-trigger baseline in at least one primary ROI, and up to 18 (17.2; 0.9) in at least one investigated AAL parcel. During the recognition phase, up to 17 (mean 17.0; SD 0.0) of 20 participants showed significant beta desynchronization in at least one primary ROI, and up to 17 (16.37; 1.3) in at least one investigated AAL parcel. Analysis of beta power relative to baseline revealed widespread decreases, strongest in the left hemisphere over frontal, parietal, and temporal areas during both encoding and recognition (Fig. 2).

### 3.3 Lateralization

#### 3.3.1 SME – Regions of interest

In the first session, analyzing only encoding data with subsequently recognized words yielded the highest LI of 0.34 in the IFGT with 84.6% of participants with beta synchronization (*n* = 13) showing LIs > 0.1, however did not reach statistical significance already before FDR-adjustment (*p* = 0.08, bootstrap statistics). Second highest was achieved by the PreCG, but did not achieve significance either (LI mean = 0.29, 71.4% of *n* = 14 with LI > 0.1, p = 0.08). The remaining ROIs showed lower and also non-significant results. In the second session, highest mean LIs were achieved in the STG (mean 0.32, 60% of *n* = 15, p = 0.08) and SMG (mean 0.27, 62.5% of *n* = 16, *p* = 0.18).

#### 3.3.2 SME – Exploratory analysis

In the first session, the inferior parietal gyrus (IPG) and the medial superior frontal gyrus (SFGM) showed highest LIs (IPG: mean 0.48, 78.6% of n = 14, *p* = 0.029, uncorrected; SFGM: 0.26, 83.3% of *n* = 12, *p* = 0.033, uncorrected). In the second session, only the middle temporal gyrus (mean 0.43, 73.3% of n = 15, *p* = 0.021, uncorrected) showed significant lateralization, followed by the IPG (0.39, 70.6% of *n* = 17, *p* = 0.06, uncorrected) and SFGM (0.13, 66.7% of n = 15, *p* = 0.07, uncorrected).

#### 3.3.3 Encoding – Regions of interest

**Fig. 3:**
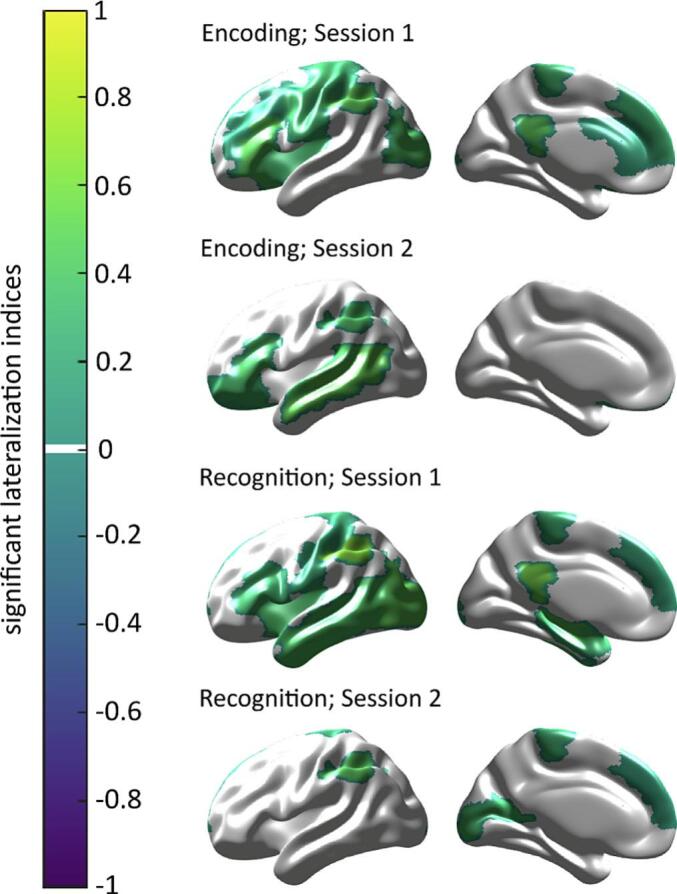
Significant LIs for each condition and session: Only regions which exhibited significant LIs are shown. Left lateralization is assumed for LIs > 0.1, right lateralization for LIs < −0.1. When looking at significant results, successful left lateralization is evident. However left lateralization was more widespread in the first session than in the second, and did not appear in all ROIs. High LI values are particularly evident in the IFGT. Additionally, the IPG, which wasn't an ROI, appears to have been well lateralized. ROI - regions of interest, LI - laterality index, IFGT - inferior frontal gyrus pars triangularis, IPG - inferior parietal gyrus.

**Fig. 4:**
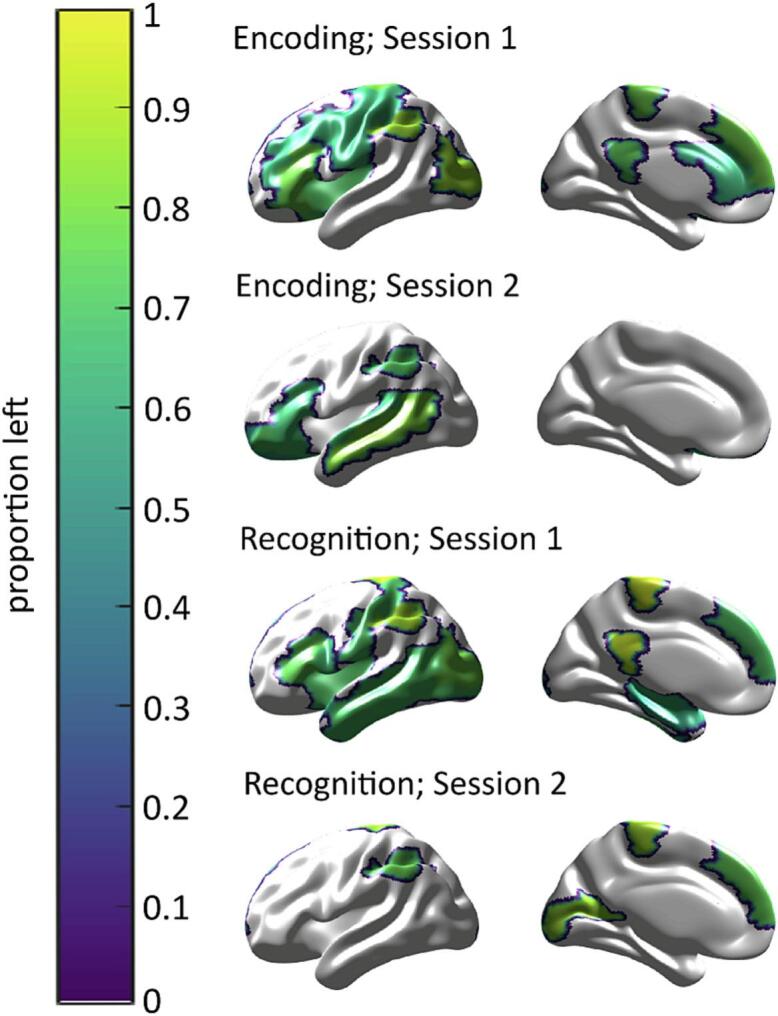
Proportion of left lateralization for each condition and session: Only regions which exhibited significant LIs are shown. When looking at significant results, the majority of participants exhibited left lateralization. In particular, the IFGT demonstrated the highest rate of lateralization among the ROIs. Furthermore, the IPG, which was not an ROI, exhibited a high percentage of left lateralization. ROI - regions of interest, LI - laterality index, IFGT - inferior frontal gyrus pars triangularis, IPG - inferior parietal gyrus.

During the first session, the IFGT (mean 0.49; 95% CI 0.23–0.75; *p* < 0.05 FDR adj.) showed the highest LI among significantly lateralized ROIs, followed by the PreCG (0.35; 0.05–0.66; *p* < 0.1). Of participants with significant beta desynchronization, most were left-lateralized in the IFGT (79%) and the PreCG (57%) (Fig. 3, Fig. 4).

During the second session, the STG (0.43; 0.12–0.73; p < 0.1) showed the highest LI, followed by the IFGT (0.40; 0.08–0.72; p < 0.1), although the FDR-adjusted *p*-values were no longer significant. Among participants with significant beta desynchronization, 61% showed left lateralization in the STG and IFGT (Fig. 3, Fig. 4).

For details, see Table 1.

**Table 1:** Encoding - ROI.

**Region**	**Beta Desynchronization (n)**	**Mean LI**	**LI > 0.1**	**p-value**	**p-value (FDR-adj.)**
**Session 1**					
IFGO	14	0.32	57%	0.101	0.202
IFGT	14	0.49	79%	0.005	0.030
SMG	13	0.07	54%	0.612	0.734
ANG	13	0.23	54%	0.237	0.356
STG	14	0.06	50%	0.754	0.754
PreCG	14	0.35	57%	0.029	0.087

**Session 2**					
IFGO	18	0.13	50%	0.504	0.504
IFGT	18	0.40	61%	0.023	0.069
SMG	18	0.18	56%	0.321	0.504
ANG	17	0.20	59%	0.364	0.504
STG	18	0.43	61%	0.015	0.069
PreCG	18	0.11	56%	0.491	0.504

Table 1: Encoding session 1 and 2:

Beta desynchronization counts per ROI with mean LIs, LIs >0.1 (indicating left lateralization), and p-/FDR-adjusted *p*-values for the encoding condition of each session.

ROI – regions of interest, LI - laterality index, IFGO - inferior frontal gyrus pars opercularis, IFGT - inferior frontal gyrus pars triangularis, SMG - supramarginal gyrus, ANG - angular gyrus, STG - superior temporal gyrus, PreCG - precentral gyrus.

**Fig. 5:**
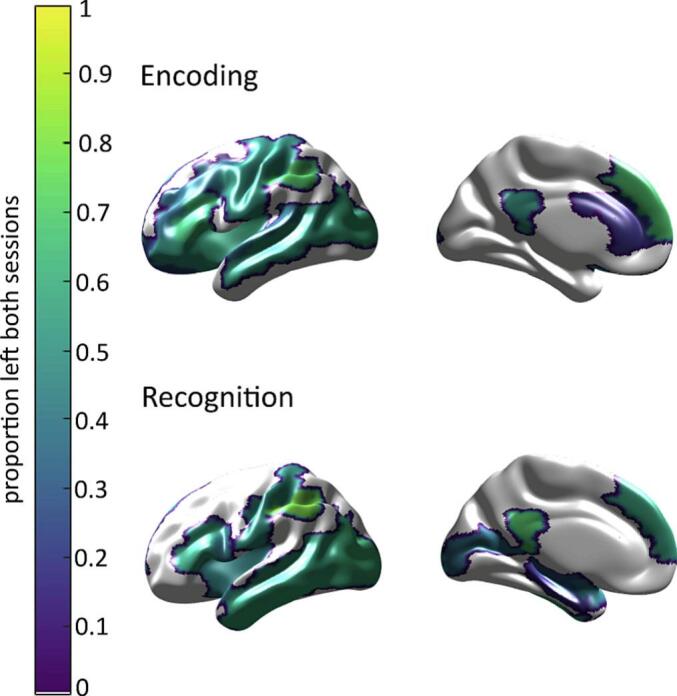
Proportion of participants with left lateralization in both sessions for each condition: Only regions with significant results in at least one session are considered. The IFGT and PreCG exhibited the highest percentages of concordant left lateralization in the ROIs, yet still lower percentages than expected. The high proportions achieved by the PCL and IPG, which are not ROIs, are also noteworthy. ROI - regions of interest, IFGT - inferior frontal gyrus pars triangularis, PreCG - precentral gyrus, IPG - inferior parietal gyrus, PCL - paracentral lobule.

When examining concordant lateralization during encoding across both sessions, the IFGT (proportion concordant lateralization: 64%) outperformed the STG (36%) and PreCG (50%) (Fig. 5).

#### 3.3.4 Encoding - exploratory analysis

In the first session of the encoding condition, exploratory analysis identified the inferior parietal gyrus (IPG; 0.49; 0.19–0.79; *p* = 0.01 not FDR-adj.) with 86% left lateralization, the medial superior frontal gyrus (SFGM; 0.28; 0.17–0.39; *p* < 0.005), the paracentral lobule (PCL; 0.32; 0.05–0.59; *p* < 0.05) both with 79% and the posterior cingulate gyrus (PCC; 0.47; 0.13–0.81; p < 0.05) with 75% left lateralization. In the second session, the IPG (0.40; 0.03–0.76; *p* < 0.05) with 72% alongside the middle temporal gyrus (MTG; 0.51; 0.25–0.77; *p* < 0.005) with 82% showed strong left lateralization (Fig. 3, Fig. 4).

Across both sessions, left lateralization during encoding was observed most strongly in the IPG and PCL (both 71%), followed by the SFGM (54%), MTG (50%), and PCC (50%) (Fig. 5).

#### 3.3.5 Recognition – Regions of interest

During the first session of the recognition phase, uncorrected significant lateralization was observed in the IFGT (0.33; 0.03–0.63; *p* < 0.05 not FDR-adj.) with 76% lateralization, IFGO (0.30; 0.04–0.57; p < 0.05) with 53% lateralization, and PreCG (0.21; −0.01 - 0.43; *p* < 0.1) with 65% lateralization, but none survived FDR correction (Fig. 3, Fig. 4).

In the second session, the PreCG (0.25; −0.02–0.52; p < 0.1 not FDR-adj.) showed modest left lateralization with 59%, though this effect also did not remain significant after FDR adjustment (Fig. 3, Fig. 4). For a detailed overview, see Table 2.

**Table 2:** Recognition - ROI.

Region	Beta Desynchronization (n)	Mean LI	LI > 0.1	*p*-value	p-value (FDR-adj.)
**Session 1**					
IFGO	17	0.30	53%	0.041	0.134
IFGT	17	0.33	76%	0.045	0.134
SMG	17	0.18	41%	0.224	0.256
ANG	15	0.18	53%	0.256	0.256
STG	17	0.22	47%	0.216	0.256
PreCG	17	0.21	65%	0.067	0.134

**Session 2**					
IFGO	17	0.13	53%	0.487	0.588
IFGT	17	0.19	53%	0.231	0.588
SMG	17	0.19	53%	0.329	0.588
ANG	17	0.14	47%	0.490	0.588
STG	17	0.09	53%	0.616	0.616
PreCG	17	0.25	59%	0.088	0.528

Table 2: Recognition session 1 and 2:

Beta desynchronization counts per ROI with mean LIs, LIs >0.1 (indicating left lateralization), and p-/FDR-adjusted p-values for the recognition condition of each session.

ROI – regions of interest, LI - laterality index, IFGO - inferior frontal gyrus pars opercularis, IFGT - inferior frontal gyrus pars triangularis, SMG - supramarginal gyrus, ANG - angular gyrus, STG - superior temporal gyrus, PreCG - precentral gyrus.

Concordant lateralization during recognition was observed most strongly in the PreCG (63%), IFGT (56%) and the IFGO (38%) (Fig. 5).

#### 3.3.6 Recognition – Exploratory analysis

In the first recognition session, exploratory analysis identified significant left lateralization in the PCL (0.27; 0.08–0.45; p < 0.05) with 94%, the PCC (0.59; 0.37–0.81; p < 0.005) with 93%, the IPG (0.64; 0.47–0.80; p < 0.005 not FDR-adj.) with 88%, and in the MTG (0.41; 0.12–0.70; p < 0.05) and SFGM (0.16; 0.06–0.27; p < 0.005) both with 71% left. In the second session, the PCL (0.28; 0.15–0.40; p < 0.005) with 88%, the IPG (0.46; 0.14–0.79; p < 0.05) and SFGM (0.13; 0.05–0.22; p < 0.05) both with 76%, again showed significant lateralization (Fig. 3, Fig. 4).

Concordant recognition-related lateralization across both sessions was observed in the PCL (81%), IPG (80%) and PCC (70%), whereas fewer participants showed concordant lateralization in the MTG (56%) and SFGM (56%) (Fig. 5).

### 3.4 Concordant lateralization

Participants who showed concordant lateralization in both encoding and recognition within a single session exhibited clear left lateralization across frontal, temporal, and parietal regions.

**Fig. 6:**
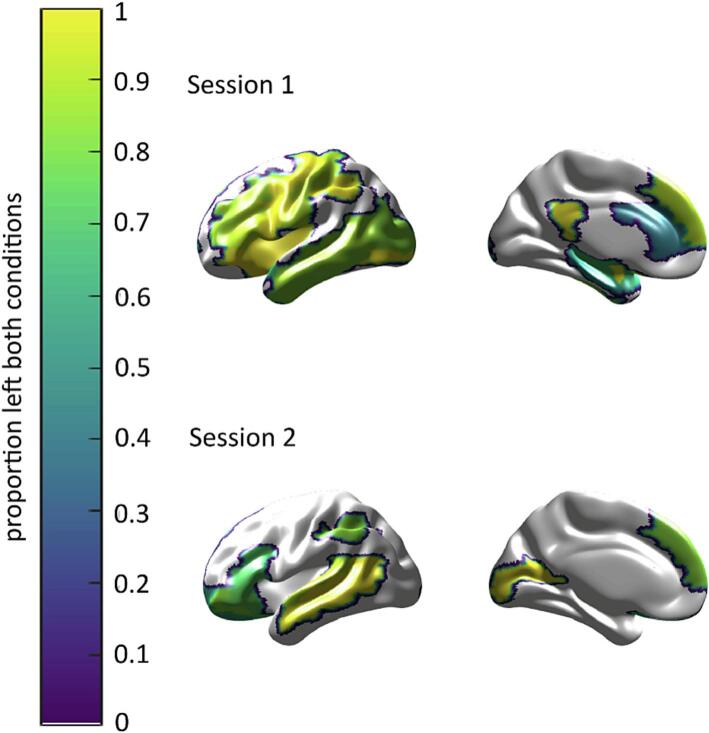
Proportion of participants with concordant left lateralization in both conditions for each session: Only regions with significant results in at least one session are considered. The high proportions of left lateralization in the frontal and precentral ROIs during the first session are particularly noteworthy. Additionally, the IPG, PCL, PCC, SFGM and MTG, although not being ROIs, exhibited very high proportions of concordant left lateralization between conditions. ROI - regions of interest, IPG - inferior parietal gyrus, PCL - paracentral lobule, PCC - posterior cingulate cortex, SFGM - medial superior frontal gyrus, MTG - medial temporal gyrus.

In the first session, 9 of 10 participants with concordant IFGT lateralization were left-lateralized (n 10; p enc FDR-adj. < 0.05); no other ROI survived FDR correction, with the PreCG (8; 100%; p enc FDR-adj. < 0.1) coming closest (Fig. 6). In the second session, the STG (9; p enc FDR-adj. < 0.1) with 89% and the IFGT (11; p FDR-adj. < 0.1) with 73% showed the strongest concordant left lateralization, but neither survived FDR adjustment (Fig. 6). For details, see Table 3.

**Table 3:** Concordant lateralization - ROI.

Region	Concordant (n)	Concordant left	p-value encoding	p-value (FDR-adj.) encoding	p-value recognition	p-value (FDR-adj.) recognition
**Session 1**						
IFGO	5	100%	0.101	0.202	0.041	0.134
IFGT	10	90%	0.005	0.030	0.045	0.134
SMG	9	67%	0.612	0.734	0.224	0.256
ANG	7	57%	0.237	0.356	0.256	0.256
STG	6	67%	0.754	0.754	0.216	0.256
PreCG	8	100%	0.029	0.087	0.067	0.134

**Session 2**						
IFGO	11	64%	0.504	0.504	0.487	0.588
IFGT	11	73%	0.023	0.069	0.231	0.588
SMG	13	62%	0.321	0.504	0.329	0.588
ANG	10	60%	0.364	0.504	0.490	0.588
STG	9	89%	0.015	0.069	0.616	0.616
PreCG	13	69%	0.491	0.504	0.088	0.528

Exploratory analysis in the first session revealed perfect (100%) concordant left lateralization in the IPG (11; p rec not FDR-adj. < 0.0001), PCL (11; p rec < 0.05) and PCC (9; p rec < 0.005) and slightly less in the SFGM (10; p enc < 0.005) with 90% and MTG (7; p rec < 0.05) with 86%.

In the second session, the MTG (10; p enc < 0.005) and PCL (10; p rec < 0.005) again showed strong concordant lateralization (both 100%), with the SFGM (12; p rec < 0.05) and IPG (12; p rec < 0.05) both with 83% slightly less so (Fig. 6).

## 4 Discussion

The presented study aimed to investigate whether lateralized beta desynchronization can be observed during a verbal SME paradigm, which largely addresses receptive language processing. Furthermore, the ability to lateralize the language-dominant hemisphere as a potential addition to the testing battery for presurgical language lateralization.

The subsequent memory effect and beta desynchronization were successfully employed to facilitate lateralization of language. A widespread beta desynchronization, with the most pronounced decrease occurring in the left hemisphere was demonstrated on the group level.

Particularly in the inferior frontal and the parietal region, lateralization was successful. ROIs based on previous research were successfully lateralized in 79% (IFGT, encoding 1) of participants at most, although the exploratory analysis revealed stronger lateralization with highest values found in the IPG (88%, recognition 1), PCC (93%, recognition 1) and PCL (94%, recognition 1).

The established language lateralization paradigm for MEG proposed by Papanicolaou, a recognition memory task, bears resemblance to SME (Breier et al., 2001; Papanicolaou et al., 2004; Papanicolaou et al., 2006). The methodology employed here is similar, in that participants are presented with a list of words, which they are required to either memorize (Papanicolaou) or rate (our study). Subsequently, the same items are presented again but intermixed with new words. Participants are then asked to show or rate their recognition of the presented stimuli (Otten and Rugg, 2002; Papanicolaou et al., 2006). In contrast to Papanicolaou's paradigm however, MEG recordings of the participants are also performed during the encoding and not only the recognition phase (Paller and Wagner, 2002; Papanicolaou et al., 2004; Kim, 2011). Furthermore, originally, dipole analysis on evoked fields was used to determine lateralization.

### 4.1 Reliability

By repeating the measurement within 7–10 days, we evaluated the reliability of SME for language lateralization. Overall, reliability was limited in the predetermined ROIs with highest values for concordant lateralization in both sessions in the IFGT (64%, encoding). Exploratory analysis showed better results, primarily within the PCL and IPG, which exhibited values reaching up to 81% and 80% of concordant left lateralization respectively in both sessions. The observed performance decline may be due to one or more of the following:

Analysis of reaction time and error rates revealed that participants were faster but made significantly more mistakes during the second session. As the order of word lists was randomized, a potential higher difficulty level during the second session can be excluded as a reason for the declined performance. We hypothesize that the participants of our study did not contemplate the words and were instead just instantaneously recalling already stored information, which is reflected by the very fast reaction times. It is established that if a task can be completed with minimal difficulty, there will be less cortical activation (Hill and Schneider, 2012). The process of contemplating the word may necessitate more activity than simply instantaneously recalling information that has already been stored, which appears to have been the predominant mechanism in this instance. Furthermore, there is evidence to suggest that slower reaction times lead to or are caused by an increase of cognitive control and a longer memory search, which may result in an improved performance relative to the outcome observed in our study (Nyhus, 2018).

Thus, the performance decline and subsequently limited reliability may be attributed to reduced motivation as the task may have been too simple for the healthy and young participants. Nevertheless, it is possible that patients with potentially reduced performance may find these tasks more challenging and thus not encounter the same issue.

In some cases, encoding and recognition yielded different lateralization results. We hypothesized that this may be due to limited data quality or task engagement, rather than differing involvement of hemispheres. Correspondingly, when the analysis was limited to those participants with concordant encoding-recognition results, a unanimous lateralization to the left hemisphere was evident.

### 4.2 Lateralization and clinical use

As supported by the literature, we postulated that the inferior frontal gyrus (IFG) would demonstrate robust lateralization and reliability, a hypothesis that our data provides further evidence for (Kim, 2011; Herfurth et al., 2022). Lateralization was successful in the predetermined ROIs with the strongest lateralization and reliability achieved by the IFGT. Numerous studies have found a relation between the encoding of stimuli and a corresponding decrease in beta power (Hanslmayr et al., 2012; Meeuwissen et al., 2011). If the chosen stimuli were words, the decrease in beta power was found in the regions associated with word processing such as the (left) inferior prefrontal cortex (Meeuwissen et al., 2011; Hanslmayr et al., 2012). Consequently, it is not surprising that our SME paradigm would yield similar outcomes regarding beta desynchronization.

However, the results show considerably lower lateralization compared to those observed during the VG paradigm of Herfurth and colleagues (Herfurth et al., 2022). Additionally the ROIs ANG, SMG and STG, or Wernicke's area which were presumed to be significantly implicated due to its involvement in the study conducted by Papanicolaou, exhibit even less discernible lateralization than the IFGT and IFGO or Broca's area (Papanicolaou et al., 2006). The exploratory findings related to the IPG, however, merit particular attention due to its excellent lateralization and its proximity to Wernicke's area.

The studies conducted by Papanicolaou et al. were only examining the recognition part of a paradigm reminiscent of SME. Additionally they were using a single dipole model for source localization, which yields single, point-like sources fitted to each data sample of the evoked response (Papanicolaou et al., 2004; Papanicolaou et al., 2006). As localizations in their study were observed in a broader temporoparietal region, it seems reasonable to suggest that the area in question may have included the IPG.

Additionally, a range of studies have demonstrated that beta desynchronization is especially associated with expressive motor language processes (Weiss and Mueller, 2012). When words are generated, a strong beta desynchronization is therefore expected to occur stronger in left frontal than in parietal regions (Singh et al., 2002; Fisher et al., 2008; Herfurth et al., 2022). Furthermore, it has been established that VG is capable of displaying both receptive and expressive language processing, while some paradigms reminiscent of SME only show the receptive processing of language (Pirmoradi et al., 2010; Papanicolaou et al., 2019). Our results show that beta desynchronization also occurs in SME, a receptive paradigm, suggesting that beta desynchronization is not specific for expressive motor language processes.

Although the IFGT demonstrated the most reliable hemispheric lateralization in our data, it was not possible to obtain unambiguous results in all participants. Furthermore, it remains unclear how the results would differ if the data had been obtained from patients rather than healthy subjects, in whom reorganization processes due to a lesion may result in a more complex (e.g. bilateral) language lateralization and determination thereof. It has been shown that during periods of slow tumor growth, compensatory mechanism emerge, initially in the surrounding areas, then subsequently extending to the opposite side (Nieberlein et al., 2023). This long-term reorganization may be essential for the preservation of language functions after surgery, though it is anticipated that minimal additional reorganization will occur if a substantial amount of reorganization has already taken place prior to the surgical intervention (Nieberlein et al., 2023). Thus, it is imperative to exercise particular caution when assessing certain patient groups such as aphasic patients.

Consequently, research suggests that MEG language tests are excellent for screening, though not yet a replacement for Wada tests (Hirata et al., 2010; Herfurth et al., 2022; Noorizadeh et al., 2024). Nevertheless, they serve as a valuable instrument for identifying stimulation sites and have the potential to reduce the time required for invasive language mapping (Hirata et al., 2010; Herfurth et al., 2022; Noorizadeh et al., 2024).

In addition, it is important to note that there are strategies to potentially improve performance of SME language lateralization. Modifications aimed at increasing the difficulty of the task may yield more reliable results, as task-engagement can be harmed by its simplicity (Hill and Schneider, 2012; Nyhus, 2018). The analysis should be focused exclusively on patients that demonstrate beta desynchronization. Furthermore, clinical use of the results may be limited to concordant lateralization under the same paradigm conditions (enc-enc, rec-rec) and when concordant lateralization is observed within the different conditions of the same session (encoding session 1 - recognition session 1).

Language processing is a complex mechanism involving a diverse spectrum of frequency bands (Coolen et al., 2024; Ervin et al., 2024). A potential further approach to improve reliability therefore may be to integrate alterations of other frequency bands as well as their dynamics over time (Coolen et al., 2024).

### 4.3 Networks related to language

#### 4.3.1 Working memory and inner speech

The regions found in the exploratory analysis of our study – MTG, IPG, SFGM, PCC, and PCL as well as the predefined ROI PreCG - have been shown to be involved with complex language and working memory mechanisms.

The beta desynchronization in the PreCG lateralized well in the majority of participants. As the PreCG is involved in motor function (Banker and Tadi, 2025), the strong lateralization in a receptive language paradigm may be surprising. However, there is existing data to support the assumption that precentral involvement in language processing may be due to its role within the semantic control network (SCN) and verbal working memory (Bourguignon and Gracco, 2019). There is strong evidence to suggest that during involvement of working memory, regions are activated, that would be used if the task was to be carried out (Postle, 2006). This can be described as a “rehearsal” or a form of inner speech that consequently activates the precentral gyrus (Postle, 2006; Pratts et al., 2023). This activation may also have the function of surveilling information and rival response options (Bourguignon and Gracco, 2019).

Evidence suggests extensive language networks, as identified in the exploratory analysis of this study. Furthermore, networks related to working memory appear to be overlapping with language areas. There are findings that support the connection of the MTG with cognitive control of semantic memory during language tasks (Bourguignon and Gracco, 2019). Additionally, the MTG seems to be involved with the (spontaneous) simulation of inner speech (Pratts et al., 2023).

As research indicates that the parietal lobe, particularly the inferior parietal lobe, is a region where social cognitive, memory, attention and language processing converge, it is probable that our paradigm, which requires participants to pay attention while rating given words and to remember them afterwards, involves this region (Bzdok et al., 2016; Numssen et al., 2021). The IPG is also associated with postretrieval control in working memory, such as establishing criteria for decision-making and surveilling the outcome of memory retrieval (Nyhus, 2018).

Additional studies found that the superior frontal cortex or Brodman's Area 8, which includes the SFGM, also takes part in the “active maintenance” process of working memory (Postle, 2006). Khan et al. found that the stimulation of the medial PFC leads to a decrease in forgetting and an increase in beta desynchronization in fronto-parietal areas, which further supports these findings (Khan et al., 2024). Furthermore, the left superior frontal gyrus is associated with inner speech (Pratts et al., 2023).

Our findings also show the posterior Cingulum (PCC) to be relevant when examining language processes. The PCC is associated with “tip of the tongue” phenomena, when recalling names (Kozlovskiy et al., 2017). Additionally, it is also known to be important for inner speech (Pratts et al., 2023).

Moreover, the PCC has been shown to be connected with the paracentral lobule (PCL), which is thought to be due to anticipation of motor processes such as a button press (Sweet et al., 2008). Furthermore, there is evidence to suggest that the PCL plays a significant role in regulating brain dynamics (Wang et al., 2025; Banker and Tadi, 2025). However, why the PCL shows strong lateralization in SME remains unclear.

### 4.4 Limitations

For evaluation of lateralization, we assumed that right-handed participants have left-hemispheric dominance for language. Although there is of course a strong association, even in healthy participants this does not always apply (Rasmussen and Milner, 1977). It has therefore been debated whether the assumption, that healthy right-handed participant equals left-hemispheric lateralization should continue to be made (Springer et al., 1999). Nevertheless, the aforementioned assumption remains valid in the overwhelming majority of cases involving healthy individuals (Springer et al., 1999). For future studies in healthy participants, additional lateralization methods, such as fMRI, transcranial magnetic stimulation (TMS) or functional transcranial doppler (fTCD) could support the definition of a robust reference standard. Ultimately, comparison to direct cortical stimulation and functional outcomes of surgery near identified regions represents the most relevant reference standard.

Furthermore, the representation of language shows higher variability in the presence of pathologies, e.g. atypical lateralization is reported significantly more often (Janecek et al., 2013). As this study is focused on healthy individuals, it remains unclear whether SME and beta desynchronization adequately reflects such variability in patients. Moreover, as previously discussed, the limited reliability of the second session should be considered. Follow-up studies are necessary to determine whether this limitation stems from the paradigm's setup or from the paradigm itself.

## 5 Conclusion

Significant lateralized beta desynchronization also occurs during receptive language processing, specifically in the context of the SME paradigm employed here. Next to language related areas, the precentral gyrus shows considerable involvement despite the absence of motor task components.

Clinically, a notable advantage of the SME paradigm is its capacity to evaluate not only receptive language function and task performance but may also provide insight into verbal memory. Consequently, it may fulfill an additional function of the Wada test (Hirata et al., 2010).

It is important to acknowledge the significance of recording during both conditions, encoding and recognition, as this approach has yielded particularly valuable data, but is a deviation of the original SME paradigm, established by Papanicolaou (Papanicolaou et al., 2006; Papanicolaou et al., 2004).

SME showed lateralization in the same ROIs as the VG paradigm, albeit to a lower degree. Subsequently combined paradigms should be encouraged and researched further.

In line with the SME paradigm, MEG recordings should be conducted during both encoding and recognition phases. If beta desynchronization is significant, lateralization concordance between phases should be investigated as this method seems to yield the most reliable data. However, if the lateralization fails or exhibits variation across conditions, the testing battery should be augmented with additional assessments.

Moreover the use of different methods of lateralization such as EEG, MEG, fMRI and Wada-testing should be deployed following a structured algorithm from least invasive to most invasive, which should be used only when the previous results should turn out to be ambiguous (Aydin et al., 2015; Papanicolaou et al., 2018).

## Data and code availability

The datasets generated and analyzed during the current study are not publicly available due to the requirement for a formal data sharing agreement but are available from the corresponding author on reasonable request.

## Author Contribution

EM was responsible for conceptualization, methodology, investigation, data curation, project administration, and writing the original draft. PRV was involved in investigation and data curation. NMV participated in validation and visualization. MK participated in data curation. AD, HH, DD and OS provided the resources. SR was responsible for conceptualization, methodology, software, formal analysis, validation, supervision, and funding acquisition. All authors contributed to the article and approved the submitted version.

## Funding

This work did not receive any specific grant from funding agencies in the public, commercial, or not-for-profit sectors. Institutional support was provided by the Department of Neurosurgery, University Hospital Erlangen.

## Declaration of generative AI and AI-assisted technologies in the manuscript preparation process

During the preparation of this work the author(s) used (DeepL Write and OpenAI's ChatGPT, GPT-5) in order to assist in the refinement of the text. After using this tool/service, the authors reviewed and edited the content as needed and take full responsibility for the content of the published article. All scientific content, analysis and interpretations are the author's own.

Table 3 : Concordant lateralization session 1 and 2:

Concordant beta desynchronization (encoding and recognition) counts per ROI/session with percentage of left lateralization, p- and FDR-adjusted p-values.

ROI – regions of interest, LI - laterality index, IFGO - inferior frontal gyrus pars opercularis, IFGT - inferior frontal gyrus pars triangularis, SMG - supramarginal gyrus, ANG - angular gyrus, STG - superior temporal gyrus, PreCG - precentral gyrus.

## Declaration of competing interest

None of the authors have potential conflicts of interest to be disclosed.
